# 83. Infectious Outcomes of CAR T-cell Therapy: Longitudinal Follow Up and Risk Stratification

**DOI:** 10.1093/ofid/ofac492.008

**Published:** 2022-12-15

**Authors:** Michael Czapka, Peter A Riedell, Jennifer Pisano

**Affiliations:** University of Chicago Hospital, Chicago, Illinois; University of Chicago Hospital, Chicago, Illinois; University of Chicago Hospital, Chicago, Illinois

## Abstract

**Background:**

Chimeric antigen receptor (CAR) T-cell therapy targeting CD19 is increasingly utilized in the treatment of a variety of hematologic malignancies. While efficacious, this therapy is associated with prolonged cytopenias and immunosuppression. Open questions remain regarding the distribution of infections beyond the first year of therapy and infectious risk stratification. We identified a cohort of CAR T-cell recipients to address these gaps in the literature.

**Methods:**

We retrospectively analyzed adult patients who received CAR T-cell therapy at our center including their infectious outcomes up to two years following cell infusion. Descriptive statistics and Mann-Whitney testing were used when appropriate. A time-to-event analysis was made assessing time from cell infusion to first infection. Univariate Kaplan-Meier (KM) analyses with log-rank testing and cox regression analysis identified significant predictors for inclusion into a multi-variate cox model of risk of first infection.

**Results:**

Baseline characteristics of the 75 patients are listed in Table 1. Etiology of infection changed in distribution over time from predominantly nosocomial pathogens more so to respiratory viruses and pneumonia (Figure 1).

In univariate KM failure analyses, receipt of tocilizumab or development of neurotoxicity was associated with more rapid onset of first infection (log-rank p-values = 0.0361, 0.0417, respectively) (Figure 2). Multivariate cox regression demonstrated that higher neutrophil count at apheresis was inversely associated with onset of infection (hazard ratio (HR) 0.81; 95% CI .67-.98; p-value .028), while development of neurotoxicity (HR 5.51; 95% CI 1.15–26.5; p-value .033) and receipt of tocilizumab (HR 3.93; 95% CI 1.15–13.42; p-value .029) were associated with onset of infection.

Baseline patient population characteristics as described by frequency (n) and percent of total population or median and inter-quartile range (IQR). Abbreviations - CAR: Chimeric Antigen Receptor. ECOG: Eastern Cooperative Oncology Group Performance Score. DLBCL: Diffuse large B cell lymphoma. HGBL: High grade B-cell lymphoma. TFL: Transformed follicular lymphoma. MCL: Mantle-cell lymphoma. PMBCL: Primary mediastinal large B-cell lymphoma. ALL: acute lymphoblastic leukemia. MM: Multiple myeloma. IVIG: Intravenous immune globulin. ANC: Absolute neutrophil count. ALC: Absolute lymphocyte count. IgG: Immunoglobulin G levels. *Indicates missing data on 1 patient, percent relative to n of 74 for these variables. The letter "a" indicates there was one patient each with PMBCL, ALL or MM.

Abbreviations - RVI: Respiratory Viral Infection, UTI: Urinary Tract Infection, CDI: Clostridium dificile infection, CAR: Chimeric Antigen Receptor. *Indicates graphical result truncation of RVI number between 30 days and 1-year post-CAR T-cell infusion (n=24), which is the most predominant infection. “Other” infections within 30 days of cell infusion included 2 episodes of cellulitis, and one episode each of sialadenitis, Aspergillus pneumonia and empyema. “Other” infections between 30 days and 1-year post-cell infusion included 1 each of oral HSV, intra-abdominal infection, empyema, conjunctivitis, cellulitis, bacterial sinusitis and tooth abscess. “Other” infections between 1- and 2-years post-cell infusion included shingles and bacterial sinusitis (1 each).

Kaplan-Meier failure curves of proportion of patients who developed their first infection (failure) following CAR T-cell infusion by day since cell infusion. Right censoring at two years of follow up, death, or disease progression, whichever came first. A. Curves separated by receipt of tocilizumab (red dashed line) versus no receipt of tocilizumab (solid blue line) during period of follow up. Log-rank test p = 0.0361. B. Curves separated by development of CAR T-cell neurologic toxicity (red dashed line), also known as immune effector cell-associated neurotoxicity syndrome (ICANS) as documented by diagnostic criteria from American Society of Transplant and Cellular Therapy (ASTCT) versus no ICANS (solid blue line). Log-rank test p = 0.0417.

**Conclusion:**

The pattern of infections following CAR T-cell infusion changes over time, transitioning from nosocomial infections to respiratory viral infections. The multi-variate cox model of time to first infection demonstrated neutropenia at apheresis, tocilizumab receipt and development of neurotoxicity predicted more rapid onset of infection. Strategies to mitigate inflammatory complications may reduce infection.

**Disclosures:**

**All Authors**: No reported disclosures.

